# Synovial sarcoma of the vulva: a case report

**DOI:** 10.1186/1752-1947-5-95

**Published:** 2011-03-10

**Authors:** Viren Asher, Gerhard van Schalkwyk, Anish Bali

**Affiliations:** 1 Department of Obstetrics and Gynaecology, Royal Derby Hospital, Uttoxeter Road, Derby, DE22 3NE, UK; 2 Department of Pathology, Royal Derby Hospital, Uttoxeter Road, Derby, DE22 3NE, UK

## Abstract

**Introduction:**

Contrary to its name, synovial sarcoma does not arise from the synovial membrane but from multipotent stem cells and can present in any part of the body. Very few cases of vulval synovial sarcoma have been reported in the literature; we report on such a presentation. These tumors can present as painless lumps, which must be completely excised to give the best prognosis. Therefore the diagnosis of synovial sarcoma should always be kept in mind in the management of vulval masses, especially in young patients.

**Case presentation:**

We report the case of a 28-year-old Caucasian woman with synovial sarcoma of the vulva. Complete excision was possible in this case.

**Conclusion:**

We have presented a rare case of synovial sarcoma of the vulva, which can be easily confused with lipoma of the vulva. The management of this tumor requires referral to a cancer centre, with a multidisciplinary approach.

## Introduction

Synovial sarcoma is the fourth most commonly occurring sarcoma, accounting for 7-8% of all sarcomas [1]. It most frequently occurs in young adults, with up to 30% being manifested during the first two decades of life, and a median age of 13 years at presentation [2]. There is a slight male predominance.

The term synovial sarcoma was coined to denominate tumors arising near tendon sheaths and joint capsules. Despite its name, synovial sarcomas do not appear to arise from synovial membranes, but from unknown multipotent stem cells that are capable of differentiating into mesenchymal and/or epithelial structures [3] and lack synovial differentiation [4].

Only five cases of synovial sarcoma arising from the vulva have been reported in the literature [5] as summarized in table 1.

**Table 1:** Previous reported cases of synovial sarcoma of the vulva

Case no	Presentation	Treatment	Disease free survival	Reference
1 & 2	30 and 37-year-old patients presented with painless mass on vulva	Excision of the lumps	Both patients alive and well after four years of follow-up	[11]

3	33 year-old presented with painless lump in vulva	Partial radical vulvectomy with rotational flap vulvoplasty	Alive eight months post-operatively	[12]

4	50-year-old found lump following discomfort at intercourse	Initial radiotherapy followed by radical hemi-vulvectomy followed by brachytherapy	Alive at 14 months	[5]

5	33-year-old presented with painless mass on vulva	Wide local excision followed by chemotherapy	Alive at two years	[13]

## Case presentation

A 28-year-old Caucasian woman, *virgo intacta*, was referred to us, presenting with a four-month history of a left vulva swelling, which was becoming increasingly uncomfortable. There was no other gynaecological history to note, and our patient did not report masses elsewhere.

Clinical examination revealed a healthy woman with a well-defined deep mobile mass in her left labium majus. She underwent excision of the swelling under general anesthetic and the lump, which did not appear to be attached to adjoining structures, was dissected free from the underlying tissue.

**Figure 1:**
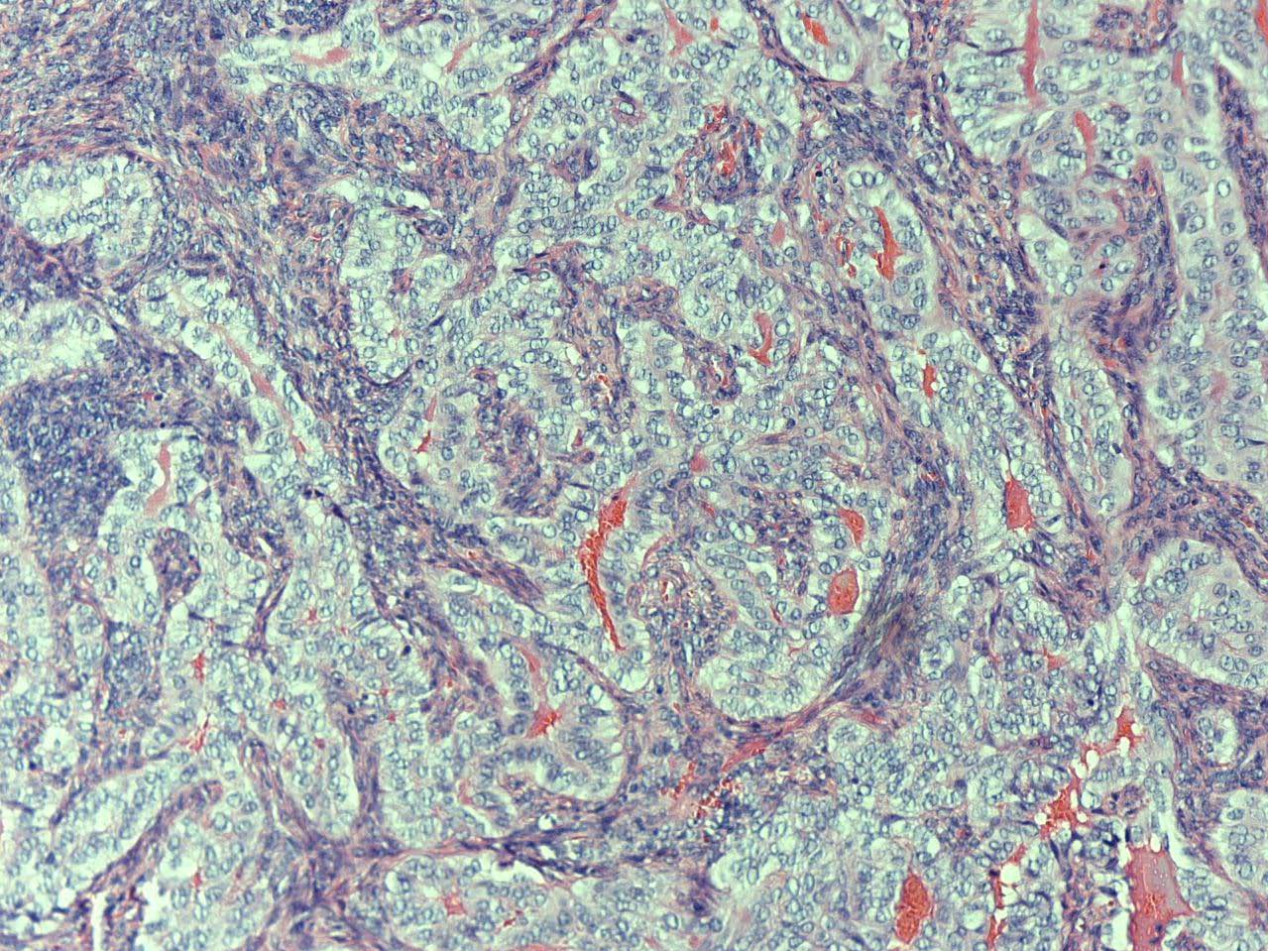
**Synovial sarcoma showing epitheloid component with glandular and/or tubular formation, surrounded by malignant spindle cell component**.

Histology demonstrated a large biphasic tumor (measuring 2.2 × 1.5 × 1.0 cm) consisting of epithelial cells and fibroblast-like spindle cells, with the presence of glandular structures and mitoses. There was also a hemangiopericytomatous pattern in some areas, with spindled and rounded cells adjacent to numerous vessels without muscular walls, and myxoid areas together with zones of definite formation of epithelial structures with large lining cells surrounded by a spindle cell matrix. Bone formation was seen focally and there was a retiform component to the tumor (figure 1). The immunohistochemical stains LCA, CD68, NSE, CD34, CAM 5.2, SMA, desmin, and S100 were negative. The keratin marker AE1/3 and CD 117 showed focal positivity and the vimentin marker was also positive. Reverse transcriptase-polymerase chain reaction performed on the RNA extracted from the tumor tissue revealed the presence of a SYT-SSX translocation. A second opinion was sought, which was in agreement with the diagnosis.

A decision to re-excise the vulval scar was taken, as there was doubt about the completeness of the first excision. A pre-operative computed tomography (CT) scan of the chest, abdomen and pelvis showed no evidence of metastatic disease. Re-excision by a consultant gynecological oncologist showed no residual tumor on histology. After discussion at our sarcoma multidisciplinary team meeting, it was felt that further adjuvant therapy was not necessary in view of the completeness of excision. We have followed up our patient with clinical examinations and a chest X-ray every three months for the last three years, and there is still no evidence of recurrence.

## Discussion

Synovial sarcoma is a soft tissue sarcoma and is divided into three subtypes: biphasic, monophasic and poorly differentiated [1]. The biphasic type contains epithelial and spindle cell elements, which occur in varying proportions, whilst the monophasic type contains only spindle cells [6]. The poorly differentiated type shares features of both the monophasic or biphasic types, and has varying proportions of poorly differentiated areas characterised by high cellularity, pleomorphism, numerous mitoses and often necrosis. All morphological subtypes are characterised by a specific t(X; 18) (p11.2; q11.2) chromosomal translocation.

It is difficult to recognize synovial sarcoma on the basis of only its histological appearance. In most cases, it can only be unambiguously identified by immunohistochemical analysis, ultrastructural findings and the demonstration of the specific chromosomal translocation mentioned above.

It most commonly presents as a slowly growing, painless mass, with the most common sites of presentation being at the extremities. The lower extremities are more frequently affected than upper extremities, often in the region of the thigh and knee. The peri-articular regions are especially affected, usually in close association with the tendon sheath, bursae and joint capsules, but they rarely involve the articular surface [6]. However, there is a broad spectrum of locations where they have presented, including head, neck, trunk, lungs, esophagus, intestine, mediastinum and retroperitoneum. The major sites of metastatic spread are the lungs and, less often, regional lymph nodes, bone and bone marrow [1].

A definitive diagnosis can only be established by an adequate tissue biopsy, but radiological investigations may be useful to characterise the tumor. On a CT scan, the tumor usually shows as a heterogenous septate mass with a mixed solid and cystic appearance; calcification can be seen in one third of them. Magnetic resonance imaging (MRI) provides a greater contrast between tumor and normal tissue and can show neurovascular or regional lymphatic involvement [7]. Radiological investigations are important in the detection of metastatic disease, as the presence of secondary spread influences both prognosis and management.

The presence of metastases, a tumor size greater than 5 cm, invasiveness, high histological grade, positive surgical margins and poor histological differentiation are all associated with adverse prognostic significance [2].

The complete surgical excision of the tumor is the treatment of choice. Often, synovial sarcoma has a pseudocapsule that allows the tumor to shell out fairly easily, giving a false sense of security that complete removal has occurred [8]. The presence of positive microscopic margins gives a greater likelihood of local recurrence and is associated with an increased risk of metastatic spread and decreased length of disease-free survival. Therefore, the surgeon should aim at obtaining negative margins, although there is no clear evidence on the extent of negative margin required [9]. Primary re-excision should be carefully considered in all patients with a gross residual tumor, as re-excision decreases the risk of local recurrence [8].

Radiotherapy appears to have a role in the treatment of patients newly diagnosed with synovial sarcoma, especially children with minimal primary tumor or following surgery [10]. Radiotherapy should improve local control of the disease but does not affect overall survival. The role of adjuvant chemotherapy in the management of synovial sarcoma remains controversial.

Future areas of investigation should include the role of molecular genetics to evaluate for possible targeted therapy, as well as the role of chemotherapy and radiation in improving prognosis.

## Conclusion

This is a rare case of synovial sarcoma of the vulva, the appearance of which can be easily mistaken for a lipoma. The overall prognosis for our patient should be good given the complete excision of the tumor. Other favorable factors affecting the prognosis include a tumor size of less than 5 cm and no evidence of metastatic spread.

## Consent

Written informed consent was obtained from the patient for publication of this case report and any accompanying images. A copy of the written consent is available for review by the Editor-in-Chief of this journal.

## Competing interests

The authors declare that they have no competing interests.

## Authors' contributions

VH was involved in pre- and post-operative care of the patient and wrote the manuscript. GVS performed the histopathological examination and made the histological diagnosis. AB performed the surgery and helped in correction of the manuscript. All authors have read, approved and contributed towards the final manuscript.
